# Development of a fluorescent immunochromatographic assay based on quantum dots for the detection of fleroxacin

**DOI:** 10.1039/d1ra03065e

**Published:** 2021-06-22

**Authors:** Qingbao Yang, Yanhua Qi, Jingming Zhou, Yumei Chen, Chao Liang, Zhanxiang Liu, Xiaoli Zhang, Aiping Wang

**Affiliations:** School of Life Sciences, Zhengzhou University Zhengzhou 450001 Henan China pingaw@126.com

## Abstract

Fleroxacin (FLE) is a broad-spectrum fluoroquinolone antibiotic widely used in animal husbandry, veterinary medicine and aquaculture. Eating animal-derived foods with FLE residues can cause allergies, poisoning or drug resistance. The water-soluble QDs (CdSe/ZnS) and anti-FLE monoclonal antibody (mAb) were used to prepare a fluorescent probe by the method of *N*-(3-dimethylaminopropyl)-*N*′-ethylcarbodimide hydrochloride (EDC) activation. The fluorescent probe was characterized by dynamic light scattering (DLS). The better bioactivity and stability of the fluorescent probe was obtained under the pH value of 8.0, the molecule molar ratio of EDC (1 : 2000) and anti-FLE monoclonal antibodies (1 : 10). The control line (C line) and test line (T line) of a nitrocellulose (NC) filter membrane were sprayed with SPA (0.05 mg mL^−1^) and FLE-OVA (1.4 mg mL^−1^) solutions with optimal concentration, respectively. A novel method of fluorescent immunochromatographic assay based on quantum dots (QDs-ICA) in this work exhibited good accuracy, reproductivity and excellent specificity under the optimal experimental conditions. Compared with the traditional method for the visual detection of FLE, the developed QDs-ICA can successfully determine FLE residues in pork meat with a better cut-off value of 2.5 ng mL^−1^. The QDs-ICA could be adapted for the rapid preliminary detection of FLE residues in pork meat for the first time.

## Abstract 2

(1) Water-soluble CdSe/ZnS QDs and an anti-FLE monoclonal antibody were used to prepare a fluorescent probe. (2) Primary rapid detection of FLE residues with visual fluorescent detection method.

## Graphical abstract

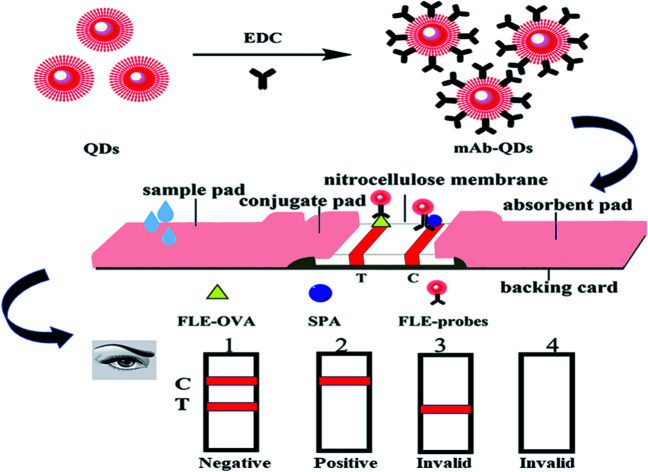

## Introduction

Fleroxacin (FLE), an organic compound (6,8-difluoro-1-(2-fluoroethyl)-1,4-dihydro-7-(4-methylpiperazino)-4-oxo-3-quinolinecarboxylic acid), is a broad-spectrum fluoroquinolone antibiotic.^1^ Different quinolones and fluoroquinolones have been applied in the treatment of animal disease around the world owing to their broad spectrum of activities for pathogenic microorganisms.^2^ Fluoroquinolones have been used in domestic animals, veterinary medicine, and aquaculture industry.^3,4^ FLE, as one of the third-generation fluoroquinolones, is harmful to public health due to residues in edible animal foods.^5^ In order to safely utilize and control the occurrence of FLE in agricultural products, it is urgent to establish a rapid, low-cost, high sensitivity and practical method for the detection of FLE residues.^6,7^

Up to now, a variety of available methods for FLE detection have been established, including enzyme-linked immunosorbent assay (ELISA),^4,8^ fluorescence-linked immunosorbent assay,^5^ high-performance liquid chromatography (HPLC) method,^9,10^ liquid chromatography-mass spectrometry (LC-MS),^11,12^ surface-enhanced Raman spectroscopy (SERS),^13^ and electrochemistry.^14,15^ These methods are popular and common due to their superiority of good specificity and high sensitivity. HPLC and LC-MS require some sophisticated instruments, maintenance, laborious sample processing, long analysis time, and skilled operators. Therefore, these instrumental methods cannot quickly screen large-scale FLE residue samples.^16,17^ In contrast, the current immunochromatographic assay (ICA) is a common and portable method for the detection of FLE residues. ICA has been widely reported and applied in environmental analysis, clinical diagnosis, and food safety determination due to its simplicity, speed, convenience and sensitivity.^18,19^

To date, various nanomaterials, including colloidal gold (CG) nanoparticles, fluorescent dye liposomes, magnetic nanoparticles, and QDs, have been widely used in the ICA as signal labels. Among these diverse nanomaterials, QDs are generally considered as excellent fluorescent probe labels for developing highly sensitive ICA. Previous research studies have reported on the use of QDs as the fluorescence label in immunoassays.^20,21^ Compared with traditional dye molecules and CG, QDs have excellent fluorescence characteristics, including a wide and continuous excitation spectrum, narrow and symmetrical emission spectrum, adjustable color, high photochemical stability, long fluorescence lifetime and high quantum yield.^22^ QDs are also excellent fluorescent label candidates because of their photoluminescence brightness, and widely applied in the life sciences, semiconductor devices, medical and health fields.^23^ QDs could improve the sensitivity of test strips compared to CG.^22^ The developed quantum dots-based immunochromatographic assay (QDs-ICA) could be adapted for the rapid preliminary detection of FLE residues in pork meat. This efficacious method provides a technical support for the comprehensive detection of veterinary drug residues and improvement of food safety.

Drug residues and illegal additives have always been the key test objects in food safety supervision. The ICA based on the antigen–antibody specific reaction is currently one of the mainstream techniques for the rapid detection of veterinary drug residues due to its advantages of simplicity, speed, and low cost. To our knowledge, the current carboxylated CdSe/ZnS QDs as fluorescent probes of ICA for the detection of FLE residues have not been reported so far. Herein, the purified monoclonal antibody and carboxylated CdSe/ZnS QDs were used to prepare the fluorescent probe (mAb-QDs) by the method of EDC activation. The development of QDs-ICA for the primary detection of FLE will be beneficial to the effectiveness of food contamination screening. Our team expects to develop a QDs-ICA that could be adapted for the rapid preliminary detection of FLE residues in pork meat.

## Materials and methods

### Materials

The water-soluble quantum dots (ZnCdSe/ZnS, QDs-COOH) with 605 nm emission wavelength were obtained from Jiayuan Quantum Dots Co., Ltd (Wuhan, China). FLE, flumequine (FLU), quinocetone (QUI), oxaquinic acid (OA), sarafloxacin (SAR), norfloxacin (NOR), ciprofloxacin (CIP), enoxacin (ENO), difloxacin (DIF), and lomefloxacin (LOM) were obtained from Dr Ehrenstorfer GmbH (Augsburg, Germany). Bovine plasma albumin (BSA) was obtained from Maokang Biotechnology Co., Ltd (Shanghai, China). Ovalbumin (OVA) was obtained from Merck KGaA (Darmstadt, Germany). *N*-(3-Dimethylaminopropyl)-*N*′-ethylcarbodimide hydrochloride (EDC·HCL) was obtained from Aladdin Biochemical Technology Co., Ltd (Shanghai, China). Staphylococcal protein A recombinant (SPA) was obtained from Yaxin Biotechnology Co., Ltd (Shanghai, China). The 96 microtiter plates were obtained from Corning Co., Ltd (Corning, USA). The anti-FLE monoclonal antibody (mAb) 5F10 was prepared in the Henan Provincial Key Laboratory of Immunobiology, Zhengzhou University. The anti-FLE ascites were purified by the method of precipitation using caprylic acid and ammonium sulfate. The nitrocellulose filter membrane (NC) CN140 was obtained from Sartorius (Gottingen, Germany). The absorbent pad and sample pad were obtained from Tongcheng Paper Products Co., Ltd (Anhui, China). The adhesive bottom plate and a card case of the test strips were obtained from Jieyi Biotechnology Co., Ltd (Shanghai, China). Water purification was performed by a Milli-Q IQ7000 instrument (Millipore, USA).

### Instruments

A BioDot CM4000 Guillotine Cutter and a BioDot XYZ3050 Dispense Platform were purchased from BioDot (BioDot, USA). A High-speed Refrigerated Centrifuge D-37520 was obtained from Sigma Laboratory Centrifuges (Sigma, Germany). An electrophoresis equipment DYCZ-24 was obtained from Liuyi Biotechnology Co., Ltd (Beijing, China). A desktop constant temperature oscillator THD-200 was obtained from Beijing YATAIKELONG Instrument Technology Co., Ltd (Beijing, China). A glass homogenizer was obtained from Shanghai Chemical Branch Laboratory Equipment Co., Ltd (Shanghai, China). An electro-thermal fanned dryer DHG-9203A was obtained from Olabo Electronic Commerce Co., Ltd (Jinan, China). The ultraviolet visible light absorption spectrum was provided by a UV-visible spectrophotometer (BioTeke, China). A multifunctional microplate reader was purchased from Molecular Device (Danaher, USA). A handheld UV lamp BG-32-A was purchased from Noted Scientific Equipment Co., Ltd (Zhejiang, China). A fluorescence test strip reader was purchased from Micro test Biotechnology Co., Ltd (Nanjing, China). A Malvern Zetasizer Nano was obtained from Malvern Equipment Co., Ltd (Malvern, England). A paper trimmer was purchased from Deli Group Co., Ltd (Ningbo, China).

### Preparation of immunolabelled probes based on QDs

**Fig. 1:**
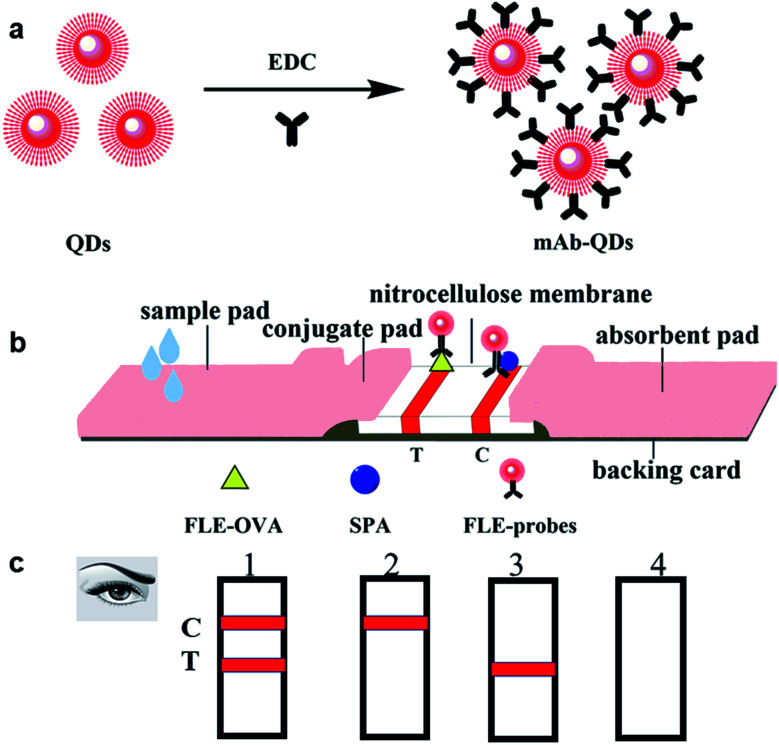
Schematic illustration of the fluorescent probes mAb-QDs and the developed QDs-ICA. (a) Synthesis of fluorescent probes mAb-QDs. (b) Preparation of QDs-ICA. (c) The QDs-ICA for FLE detection with the naked eyes. (1) Negative; (2) FLE (+); (3) and (4) invalid.

The complete antigen was prepared by coupling FLE with BSA and OVA by the carbodiimide method.^5^ FLE-BSA and FLE-OVA are used for the immunization of Balb/c mice and screening of monoclonal antibodies, respectively. For QDs-labelled fluorescent probes, anti-FLE mAb-QDs conjugates were prepared by the method of EDC activation,^24^ and a schematic illustration of the fluorescent probes mAb-QDs is shown in Fig. 1a. In brief, 10 μL of the water-soluble quantum dots (ZnCdSe/ZnS, QDs-COOH, 8 μM) with 605 nm emission wavelength was added to a 1.5 mL brown centrifuge tube. Subsequently, 1 mg EDC was dissolved in 1 mL phosphate buffer saline (PBS). The above-mentioned solution was reacted in a shaking incubator for 30 min at 25 °C. The water-soluble quantum dots were activated by EDC. The anti-FLE antibody (5F10) was first diluted by PBS (10 mM, pH 7.4), and 120 μL of the anti-FLE mAb (1.0 mg mL^−1^) solution was added into the above mixture solution. With constant gentle stirring in a shaking incubator, the reaction of the mixture solution proceeded for 3 h at 25 °C under airtight conditions. In order to block the excess carboxyl sites of free QDs, additional BSA solution (100 mg mL^−1^, 20 μL) needs to be added. The anti-FLE-mAb-QDs solution was blocked by BSA solution for 30 min. The fluorescent FLE-probes were stored in a refrigerator at 4 °C in the dark. Subsequently, considering the influence of the pH value, the amount of EDC and anti-FLE mAb on the fluorescence probes synthesis, these synthetic conditions were optimized in this paper.

### Characterization of QDs and fluorescent probes

The fluorescent probes were characterized and analyzed to determine whether the QDs and anti-FLE mAb were successfully coupled according to a previous report.^25^ The ultraviolet visible light absorption spectrum was provided by a UV-visible spectrophotometer. The fluorescence spectra of the anti-FLE mAb-QDs and pure QDs were measured by a multifunctional microplate reader. Using a Malvern particle size analyser, the size and zeta (*ζ*) potential of the anti-FLE mAb-QDs conjugates and pure QDs were obtained by dynamic light scattering (DLS). The bioactivity of the anti-FLE mAb-QDs was confirmed by ICA under a handheld UV lamp BG-32 A. Furthermore, agarose gel electrophoresis and SDS-polyacrylamide gel electrophoresis (SDS-PAGE) were performed in this study.

### Preparation of fluorescent immunochromatographic test strips

The quantum dots-based fluorescent immunochromatographic test strip was prepared according to a previous report.^26^Fig. 1b visually shows the structure and shape of the test strip. In brief, the QDs-ICA were assembled as follows: the control (C) line and the test (T) line were separated with a distance of 5 mm by a film spraying machine. Subsequently, the C line and T line of the NC membrane were sprayed with SPA and FLE-OVA solutions, respectively. The NC membrane was dried in an electro-thermal fanned dryer DHG-9203A at 40 °C for 4 h. In addition, the conjugate pad and sample pad were treated with PBS (0.01 M, pH 7.4), involving 1.0% (w/v) BSA, 0.25% (v/v) Tween-20, and 0.1% (w/v) NaN_3_. The above pre-treated conjugate pads and sample pads were dried in an electro-thermal fanned dryer DHG-9203A at 37 °C for 12 h. Ultimately, the conjugate pad, the NC membrane, the sample pad, and the absorption pad were immediately stuck on a backing card with a 2 mm overlap. In the end, using a BioDot CM4000 Guillotine Cutter, the pre-attached backing card was continuously cut into a 2.79 mm wide piece and fixed in the white card. The above-assembled QDs-ICA was securely stored in a vacuum drying oven for the next experiment.

### Rapid detection of FLE in pork meat *via* QDs-ICA

The pork meat sample was prepared according to a previous report.^27^ Briefly, the fat of the pork meat sample was first removed, and 5.0 g of the pork meat sample was chopped and homogenized by a glass homogenizer. Subsequently, 10 mL of extraction buffer (0.01 M PBS/0.154 M KCL) was added. The mixture was vortexed by a Thermo Scientific vortex mixer for 10 min, and the pork meat extraction solution was treated with centrifugation at 5000 × *g* for 10 min. Subsequently, the supernatant of the extraction solution was transferred for further detection. The FLE standard was added to the above-mentioned extraction solution to prepare different concentrations of FLE solutions. The final standard concentrations were 0, 0.001, 0.005, 0.01, 0.05, 0.1, 0.5, 1.0, 2.5, 5, and 10 ng mL^−1^. The pre-prepared fluorescent QDs-ICA was taken for the determination of the FLE standard solutions. Fig. 1c shows the illustration of the results of the developed QDs-ICA for FLE detection with the naked eyes. The sensitivity of the developed QDs-ICA was evaluated by detecting the above FLE standard solution. Corresponding to the complete disappearance of the red fluorescent band in the T-line, the lowest concentration of FLE was the cut-off value of QDs-ICA according to a previous report.^28^ Using a UV lamp BG-32-A for observing the fluorescent colour, the brightness of the T-line fluorescent band gradually faded with an increase of the concentration of FLE until the red fluorescence disappeared with the naked eyes.

## Results and discussion

### Evaluation of QDs and fluorescent probes mAb-QDs

**Fig. 2:**
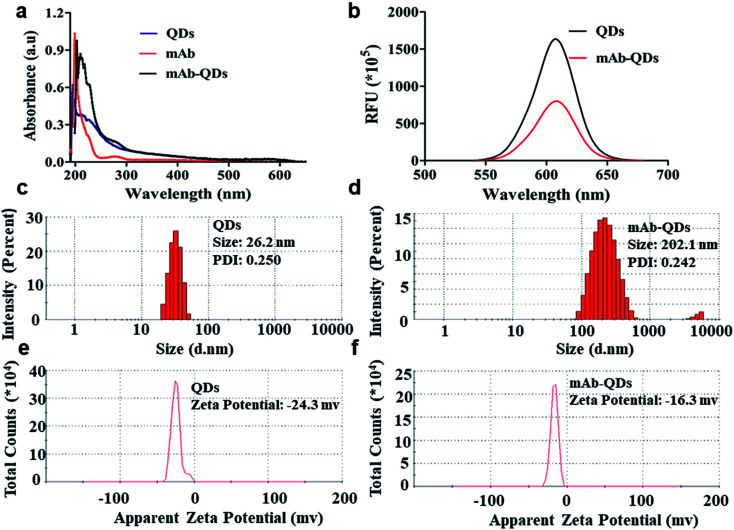
Characterization of water-soluble QDs, mAb, and fluorescent probe mAb-QDs. (a) UV/Vis absorbance spectrum. (b) Fluorescence emission spectra (Em: 610 nm). (c) and (d) Size. (e) and (f) Apparent zeta potential.

**Fig. 3:**
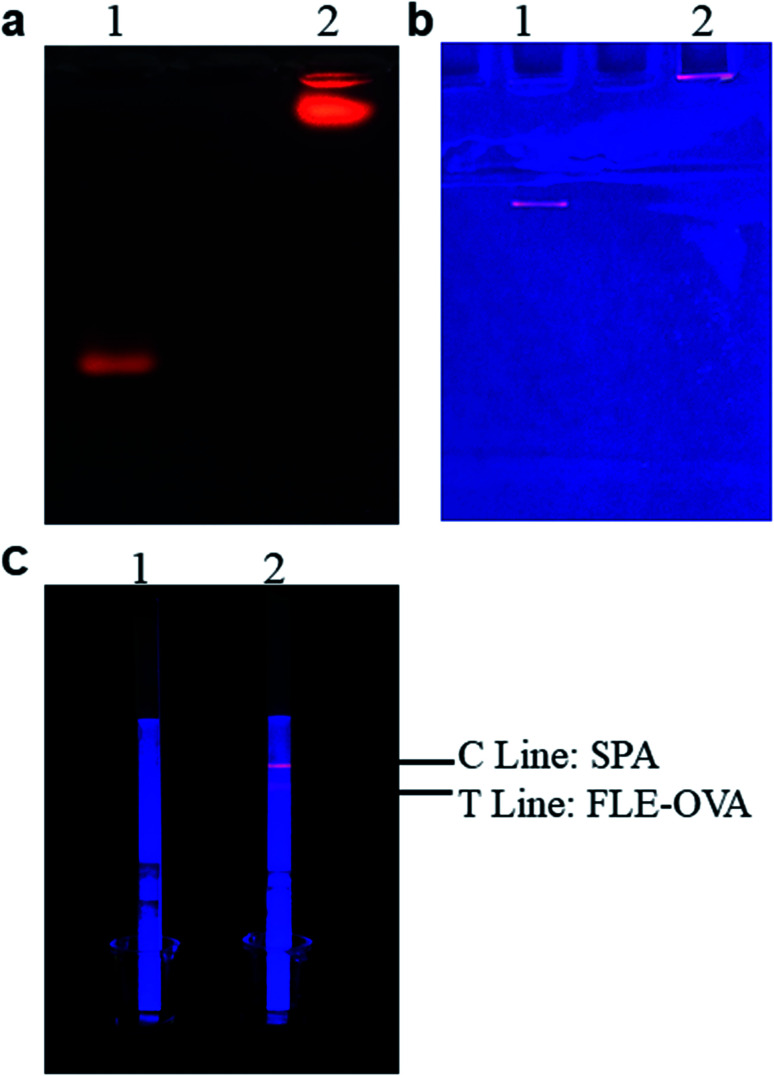
Characterization of QDs and mAb-QDs by agarose gel electrophoresis and SDS-PAGE, (1) QDs, (2) mAb-QDs. (a) Result of agarose gel electrophoresis. (b) Result of SDS-PAGE. (c) Result of original bioactivity.

As shown in Fig. 2a, the ultraviolet visible light absorption spectrum of the fluorescent probes anti-FLE mAb-QDs, the anti-FLE mAb, and the QDs were performed by a UV-visible spectrophotometer. Compared with quantum dots, the absorption value (280 nm) of the anti-mAb-QDs was significantly increased, which was due to the successful coupling of QDs and anti-FLE mAb. Furthermore, Fig. 2b shows the fluorescence spectrum of anti-FLE mAb-QDs and pure QDs. The maximum emission wavelength of the anti-FLE mAb-QDs was 605 nm, which was the same as that for QDs. However, the difference was that the fluorescence intensity of the anti-FLE mAb-QDs was slightly lower than that for QDs. It may be due to the coagulation of some quantum dots during the fluorescent probe coupling process, which had a certain influence on the fluorescence intensity. The UV-Vis absorption spectra and fluorescence intensity differences were consistent with previous reports.^29–31^ Subsequently, using a Malvern particle size analyser, the sizes of the anti-FLE mAb-QDs conjugates and pure QDs were determined by dynamic light scattering (DLS). Fig. 2c and d shows that the hydrated sizes of the anti-FLE mAb-QDs and QDs were about 202.1 nm and 26.2 nm, respectively. Compared to QDs, the size of the anti-FLE mAb-QDs significantly increased. The polymer dispersity index (PDI) value of the anti-FLE mAb-QDs was 0.242, while the PDI value of the QDs was 0.250, which showed that the anti-FLE mAb-QDs and QDs both have good dispersibility. Furthermore, the *ζ* potential of the anti-FLE mAb-QDs and QDs were performed by DLS. According to Fig. 2e and f, the *ζ* potential of the anti-FLE mAb-QDs was −16.3 mV, while the QDs was −24.3 mV. Accordingly, compared to QDs, the size and *ζ* potential value of the anti-FLE mAb-QDs had significantly changed, which was consistent with a previous report (the size increased from 10 nm to 21 nm, and the *ζ* potential changed from −41.7 mv to −32.9 mv).^32^ Furthermore, as shown in Fig. 3a and b, the agarose gel electrophoresis and SDS-PAGE results showed that the QDs run faster than anti-FLE mAb-QDs under the same conditions, which indicated that the molecular weight of the fluorescent probe anti-FLE mAb-QDs was significantly larger than that of QDs. All of the data indicated that anti-mAb-QDs were successfully prepared. In addition, the binding between the QDs and the mAb is a covalent bond, rather than electrostatic adsorption. The mAb-QD conjugate is stable and can be stored at 4 °C for five months without significant loss of activity.

### Optimization of the fluorescent probe coupling conditions

**Fig. 4:**
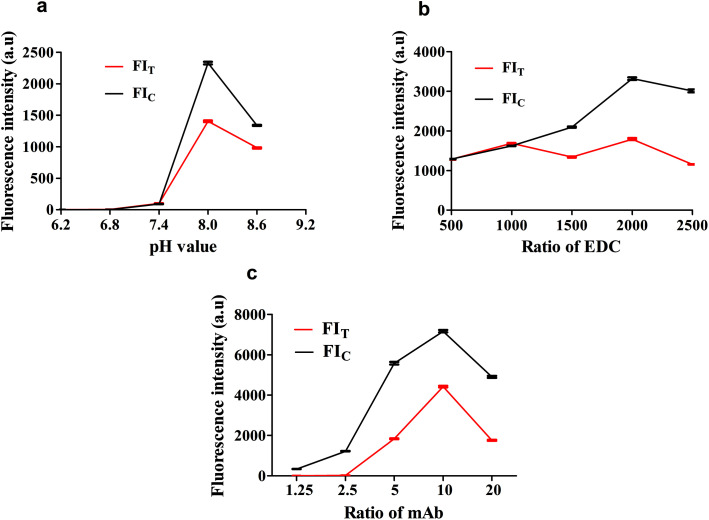
Optimization of the fluorescent probe coupling conditions. (a) Result of the pH value. (b) Result of the molar ratio of the activator EDC. (c) Result of the molar ratio of anti-FLE monoclonal antibodies. Fluorescence intensity of the control line (FI_C_), fluorescence intensity of the test line (FI_T_).

The fluorescent probe bioactivity of mAb-QDs was confirmed by ICA under a handheld UV lamp BG-32 A. Compared to pure QDs, the fluorescent probes could specifically recognize FLE-OVA and SPA according to the ICA procedure. Furthermore, Fig. 3c shows that the fluorescent probe mAb-QDs perfectly retained the original bioactivity due to ICA, with the red fluorescence appearing under the hand-held UV lamp BG-32-A (EW: 365 nm). We have optimized some synthetic factors, including the molar ratio of the anti-FLE monoclonal antibodies, EDC and pH value, which may potentially affect the bioactivity and stability of the fluorescent probe. The fluorescence intensity values of the C line and T Line were used to evaluated the optimal factor. Moreover, Fig. 4 specifically shows the results of the condition optimization. The fluorescence intensity of ICA was recorded by a fluorescent immunoassay reader. In general, Fig. 4a indicates that the fluorescence intensity values of the C line and T line first improved and reached the maximum intensity at the pH of 8.0, and then declined when the pH was 8.6. As shown in Fig. 4b, with the molecule molar ratio of EDC (1 : 2000), the fluorescence intensity values of the C line and T line showed higher values than other different molecule molar ratios. In addition, Fig. 4c indicates that the fluorescence intensity values of the C line and T line reached saturation when the molecule molar ratio of the anti-FLE monoclonal antibodies was 1 : 10. According to the above-mentioned results, the pH value of 8.0, the molecule molar ratio of EDC (1 : 2000), and the molecule molar ratio of anti-FLE monoclonal antibodies (1 : 10) were the optimization conditions of the fluorescent probe coupling in this paper.

### Optimization of several key factors of QDs-ICA

**Fig. 5:**
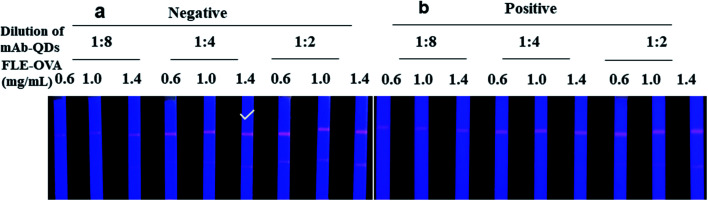
Optimization of the concentration of FLE-OVA and dilution of the fluorescent probes mAb-QDs by a method of checkerboard titration. (a) Negative, “✓” indicates the optimal concentration of FLE-OVA and dilution of fluorescent probes. (b) Positive, the concentration of FLE was 1.0 ng mL^−1^, and the fluorescence intensities of the T lines were obviously inhibited by FLE.

To obtain the best fluorescent colour development on the test lines of QDs-ICA, a series of different concentrations of FLE-OVA solution was sprayed on the T lines, and different dilutions of the fluorescent probe were used in the developed QDs-ICA by a method of checkerboard titration.^33^ The fluorescent probe and the sample solution are mixed in advance, and then dropped onto the sample pad of the QDs-ICA for subsequent detection. The volume of the fluorescent probe and the sample solution are 2 μL and 98 μL, respectively. The FLE could gradually dam the fluorescent colour development on the T line because of competitive inhibition. The result of Fig. 5a suggests that the fluorescent color development on the QDs-ICA gradually brightened for negative control as the FLE-OVA and the fluorescent probe mAb-QDs increased. In addition, Fig. 5b indicates that the fluorescence band colour development on the T line was particularly inhibited (gradually dammed) for the positive control (the concentration of FLE was 1.0 ng mL^−1^). Therefore, the optimal combinations of QDs-ICA were 1.4 mg mL^−1^ FLE-OVA and OTA-probes (dilution of 4) in this study.

**Fig. 6:**
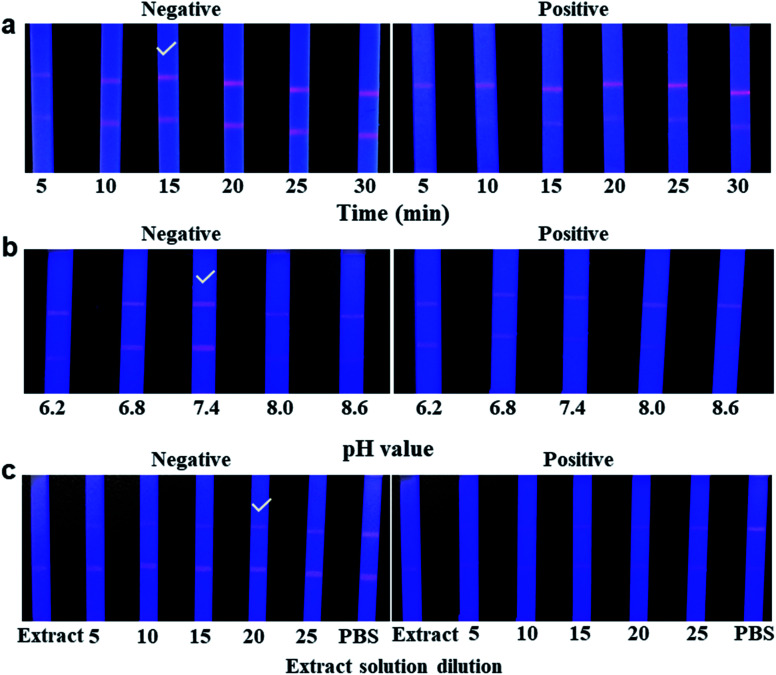
Optimization of the QDs-ICA for the detection of FLE. (a) Optimization of the reaction time for QDs-ICA. (b) Optimization of the pH value of the extract solution. (c) Optimization of the extract solution dilution. Negative, “✓” indicates the optimal result; positive, the concentration of FLE was 1.0 ng mL^−1^, and the fluorescence intensities of the T lines were obviously inhibited.

Several important factors, including the immunoreaction time, pH value, and final dilution of the pork meat extract solution, were further optimized in this study to obtain the best performance. Fig. 6a shows that the fluorescent intensity on both lines of negative control tardily increased over a time interval of 15 min. However, while extending the time of the reaction to 30 min, the fluorescence colour brightness of both lines showed no significant differences. In addition, for the positive control (FLE, 1.0 ng mL^−1^), the fluorescent colour development on the T line was obviously inhibited. Therefore, the immunoreaction time was set to 15 min for ICA analysis. The pH values of the extract solution were adjusted to 6.2, 6.8, 7.4, 8.0, and 8.6. Whether it was a negative control or a positive sample, the result of Fig. 6b showed that the pH value of 7.4 had better fluorescent intensity and competitive inhibition compared to the other pH values. In addition, the final dilution of the extract solution was investigated. Obviously, Fig. 6c indicates that the fluorescence colour of both lines is visibly brighter when the final dilution of the extract solution was gradually increased. When the extract solution was treated with a final dilution of PBS (1 : 20), the pork meat matrix effect was significantly minimized at the moment, in accordance with the fluorescence intensity and competitive inhibition being similar to the PBS control. Considering the optimum fluorescence intensity and better competitive inhibition, the final dilution of the extract solution was set to 1 : 20 in this study.

### Evaluation performance of QDs-ICA for the detection of FLE

**Fig. 7:**
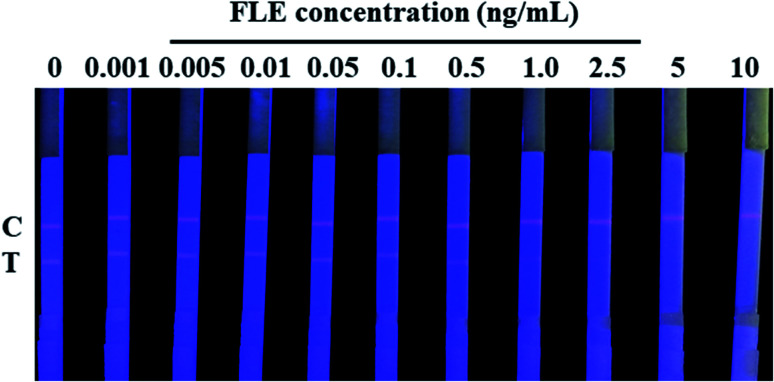
Sensitivity evaluation of the developed QDs-ICA for visual detection of FLE. The cut-off value was confirmed to be the lowest FLE concentration level, making the fluorescent intensity of the T lines completely invisible.

The cut-off value of our developed QDs-ICA for the detection of FLE in pork meat was confirmed to be the lowest FLE concentration level, making the fluorescent intensity of the T lines completely invisible according to a previously published article.^28^ After treatment with ultraviolet light, Fig. 7 shows that the brightness of the T lines fluorescent band completely disappeared as the concentration of FLE was 2.5 ng mL^−1^ with the naked eyes. Therefore, the cut-off value of QDs-ICA was 2.5 ng mL^−1^ in this study. In comparison with previous reports^4,6,34–36^ on the analytical performance of FLE detection, which are listed in Table 1, our developed QDs-ICA exhibit good sensitivity and a lower cut-off value for FLE detection in animal-derived food. This may be due to the high affinity, photoluminescence brightness and photochemical stability of the fluorescent probes. The developed QDs-ICA could be adapted for the rapid preliminary qualitative detection of FLE residues in pork meat.

**Table 1:** Comparison of previous reports on the analytical performance of FLE detection

Methods	Materials	Analyte	Sample	Sensitivity	References
ELISA and CGIA	Colloidal gold	Ofloxacin, marbofloxacin, and fleroxacin	Milk	ELISA: between 3.5 and 8.9 ng mL^−1^, CGIA: 50 ng mL^−1^ (color of the T line was invisible)	4
Dual-wavelength fluorescence detection	GSH-CdTe-QDs	Fleroxacin	Pharmaceuticals	17.93 ng mL^−1^ at 555 nm and 59.58 ng mL^−1^ at 429 nm	6
Turn-on fluorescent detection	Eu^3+^@MOF	Fleroxacin	Human serum and urine	43.91 ng mL^−1^	34
HPLC	Ionic liquid and salt	Fleroxacin and ciprofloxacin	Urine	3.12 ng mL^−1^ and 4.97 ng mL^−1^, respectively	35
MSPE-CE-UV	MSPE	Fleroxacin, gatifloxacin, lomefloxacin and norfloxacin	Milk	12.9–18.8 ng mL^−1^	36
QDs-ICA	CdSe/ZnS-QDs	Fleroxacin	Pork meat	2.5 ng mL^−1^	This work

**Fig. 8:**
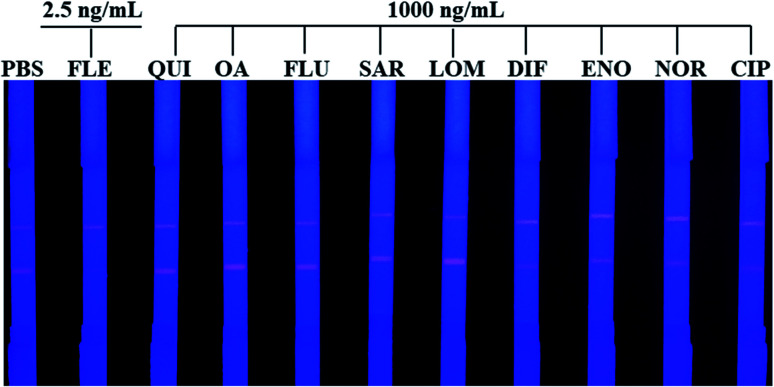
Specificity evaluation of the developed QDs-ICA for the detection of FLE. QUI, OA, FLU, SAR, LOM, DIF, ENO, NOR, and CIP were detected at a concentration of 1000 ng mL^−1^, and FLE was 2.5 ng mL^−1^.

The specificity was investigated by running QDs-ICA for the detection of several quinolones and fluoroquinolones. In brief, QUI, OA, FLU, SAR, LOM, DIF, ENO, NOR, and CIP were detected at a concentration of 1000 ng mL^−1^ by the developed QDs-ICA in this study. In addition, the negative control (PBS) and positive sample (the concentration of FLE was 2.5 ng mL^−1^) were simultaneously performed by running QDs-ICA. Compared to the negative control, Fig. 8 indicates that the fluorescent colour development on the T line showed no obvious changes in the detection of FLU, OA, SAR, NOR, CIP, ENO, DIF, LEV, and OFL even at ultrahigh concentration, whereas the red fluorescence clearly disappeared in the detection of FLE. According to the above-mentioned results, our developed QDs-ICA for the detection of FLE indicated excellent selectivity and negligible cross-reactivity with other quinolones and fluoroquinolones.

The accuracy and reproducibility were evaluated by running several different concentrations of FLE *via* the developed QDs-ICA. As shown in Table 2, the results showed that the developed QDs-ICA exhibited good accuracy and consistency. Especially, 5% false rates appeared in the FLE determination with 20 repetition tests (concentration of FLE: 2.0 ng mL^−1^). Moreover, the result of consistency was identified for other FLE-spiked levels. These observations resulted from the FLE concentration at 2.0 ng mL^−1^ being close to the cut-off value of QDs-ICA, causing the misjudgment of the detection results by naked eye. In brief, the developed ICA in this study exhibited good performance of accuracy and reproducibility.

**Table 2:** Accuracy and reproducibility of the QDs-ICA with twenty FLE-spiked pork samples

Spiked FLE (ng mL^−1^)	Visual results of the QDs-ICA (*n* = 20)			
	T line	F rate (%)	C line	F rate^d^ (%)
2.5	−−a	0	++	0
2.0	+^b^	5	++	0
1.0	+	0	++	0
0	++^c^	0	++	0

a No red fluorescence band appeared.

b The red fluorescence band was weak.

c The red fluorescence band was obvious.

d F rate = (Fn/20) × 100, and Fn was a false fluorescence band of lines.

Using the developed QDs-ICA in this study and reference method of HPLC, the reliability was evaluated by analyzing the results of several FLE-spiked pork meat samples. A series of concentrations of FLE were simultaneously detected by the two above-mentioned methods. The results shown in Table 3 indicate that our QDs-ICA could successfully distinguish these five pork meat samples at different FLE concentration levels. Furthermore, it exhibited good consistency with the reference method of HPLC. The results of these experiments showed that our reported QDs-ICA demonstrated good performance, and can be applied for the rapid and sensitive detection of FLE residues in pork meat.

**Table 3:** Comparison of QDs-ICA and HPLC in the analysis of FLE-spiked pork samples (*n* = 3)

FLE concentration (ng mL^−1^)	QDs-ICA		HPLC
	T line	C line	Results^c^ (ng mL^−1^)
100	−−a	++^b^	97.63 ± 1.31
50	−−	++	52.38 ± 1.46
25	−−	++	23.58 ± 0.92
10	−−	++	8.79 ± 0.58
5	−−	++	3.92 ± 0.54

a No red fluorescence band appeared.

b The red fluorescence band was obvious.

c Mean ± SD.

## Conclusion

In this study, the water-soluble quantum dots (ZnCdSe/ZnS) with 605 nm emission wavelength and the purified anti-FLE monoclonal antibody were used to prepare the fluorescent probe (mAb-QDs) by the method of EDC activation. A novel fluorescent ICA for the simple, portable, rapid and highly sensitive detection of FLE residues was developed. For qualitative detection of FLE residues with visual detection method, the developed QDs-ICA can successfully determine FLE residues in pork meat with the most suitable experimental conditions. Especially, the key point is that the developed QDs-ICA has a better cut-off value for the detection of FLE (2.5 ng mL^−1^) compared with a previous report. This method has improved in terms of reducing the detection time at the basic level and improving the accuracy of the detection results and efficiency, which greatly reduces the workload of the basic level detection. In brief, the developed QDs-ICA could be adapted for the rapid preliminary detection of FLE residues in pork meat.

## Author contributions

All authors contributed to the study conception and design. The experiments were performed by Qingbao Yang, Yanhua Qi and Jingming Zhou. The first draft of the manuscript was written by Qingbao Yang. All authors revised and approved the final manuscript.

## Funding

This work was financially supported by the National Key Research and Development Program of China (2019YFC1604501).

## Ethical statement

This study meets ethical standards without human participants and animals.

## Conflicts of interest

The authors declare that they have no conflict of interests or other personal relationships that could impact this research.

## Supplementary Material
